# Insight into Different Stages of Steroid Degradation in Thermophilic *Saccharopolyspora hirsuta* VKM Ac-666^T^ Strain

**DOI:** 10.3390/ijms232416174

**Published:** 2022-12-18

**Authors:** Tatyana Lobastova, Victoria Fokina, Irina Pozdnyakova-Filatova, Sergey Tarlachkov, Andrey Shutov, Marina Donova

**Affiliations:** 1 Laboratory of Bioengineering of Microbial Producers, G.K. Skryabin Institute of Biochemistry and Physiology of Microorganisms, RAS, Federal Research Center “Pushchino Scientific Center for Biological Research of the Russian Academy of Sciences”, 142290 Pushchino, Russia; 2 Laboratory of Molecular Microbiology, G.K. Skryabin Institute of Biochemistry and Physiology of Microorganisms, RAS, Federal Research Center “Pushchino Scientific Center for Biological Research of the Russian Academy of Sciences”, 142290 Pushchino, Russia

**Keywords:** thermophilic actinobacteria, steroid, catabolism, sterol degradation, *Saccharopolyspora hirsuta*, transcriptome, RT–qPCR

## Abstract

Steroids are abundant molecules in nature, and various microorganisms evolved to utilize steroids. Thermophilic actinobacteria play an important role in such processes. However, very few thermophiles have so far been reported capable of degrading or modifying natural sterols. Recently, genes putatively involved in the sterol catabolic pathway have been revealed in the moderately thermophilic actinobacterium *Saccharopolyspora hirsuta* VKM Ac-666^T^, but peculiarities of strain activity toward sterols are still poorly understood. *S. hirsuta* catalyzed cholesterol bioconversion at a rate significantly inferior to that observed for mesophilic actinobacteria (mycobacteria and rhodococci). Several genes related to different stages of steroid catabolism increased their expression in response to cholesterol as was shown by transcriptomic studies and verified by RT–qPCR. Sequential activation of genes related to the initial step of cholesterol side chain oxidation (*cyp125*) and later steps of steroid core degradation (*kstD3*, *kshA*, *ipdF*, and *fadE30*) was demonstrated for the first time. The activation correlates with a low cholesterol conversion rate and intermediate accumulation by the strain. The transcriptomic analyses revealed that the genes involved in sterol catabolism are linked functionally, but not transcriptionally. The results contribute to the knowledge on steroid catabolism in thermophilic actinobacteria and could be used at the engineering of microbial catalysts.

## 1. Introduction

Actinobacteria belonging to the genera *Mycobacterium*, *Mycolicibacterium*, *Gordonia*, *Tsukamurella*, *Rhodococcus*, *Nocardia*, and *Nocardioides* play an important role in ecologically significant processes of microbial degradation of steroids by participating in the utilization of hardly decomposable sterols of animal (cholesterol), plant (phytosterols), and fungal (ergosterol) origins [1]. Phylogenetically heterogeneous microorganisms (actinobacteria and α-, β-, and γ-proteobacteria) are capable of oxidative degradation of various steroids (sterols, cholic acids, and androstanes); however, this activity is more characteristic of actinobacteria [1,2].

The thermophilic actinobacterial genera *Amycolatopsis*, *Microbispora*, *Micromonospora*, *Thermobifida*, *Thermobispora*, *Thermomonospora*, and others have been reported to thrive on decaying organic matter [3] and are known to be beneficial in the composting process, which is characterized by their synergistic action along with bacteria and fungi. A predominance of thermotolerant actinobacteria (*Streptomyces albus* and *Streptomyces griseus*) is generally observed in thermobiotic conditions generated by preceding bacteria [4]. Moderately thermophilic actinobacteria of the *Saccharopolyspora* genus have been reported to play a role in the composting process [3]. As shown, the addition of a thermophilic compost consortium to contaminated soil enhances the bioremediation rates of pollutants, such as polycyclic aromatic hydrocarbons, petroleum, and pesticides [5], while biodegradation of steroids in waste by thermophilic actinobacteria has been little studied. Most steroids are vastly resistant to microbial degradation due to a small number of functional groups in their polycyclic structure and extremely low water solubility [1]. Various bacteria (e.g., *Comamonas*, *Mycolicibacterium*, *Pseudomonas*, *Rhodococcus*, *Sterolibacterium*, *Streptomyces*, *Sphingomonas*, etc.,) [1,6,7,8], yeasts (e.g., *Saccharomyces* and *Zygowilliopsis*) [9,10], fungi (e.g., *Aspergillus*, *Cunninghamella*, *Curvularia*, *Fusarium*, etc.,) [11], and microalgae (e.g., *Scenedesmus quadricauda*) [12] are able to transform steroids. Diverse actinobacteria can efficiently modify these hydrophobic molecules or mineralize them to CO_2_ (completely degrade), thus introducing the molecules into the carbon cycle [1,2,7,13].

Earlier, we have performed whole genome sequencing, draft assembly, and annotation of the genome of the thermophilic actinobacterial strain *Saccharopolyspora hirsuta* VKM Ac-666^T^ [14,15], which bioconverts lithocholic acid to 3α-OH- and 3-oxo-4-ene-intermediates [15,16], cholesterol to a mixture of intermediates with both 3β-OH- and 3-oxo-4-ene-structures [15], dehydroepiandrosterone (DHEA) to androst-4-ene-3,17-dione (AD) and androsta-1,4-diene-3,17-dione (ADD), AD to ADD, and 3β,7(α/β)-dihydroxy-5-ene-D-homo-lactones to the corresponding 3-oxo-4-ene-lactones and their 1(2)-dehydro analogues [17]. A distinctive feature of cholesterol bioconversion by this strain was an accumulation of a number of 26-hydroxy derivatives with both the 3β-hydroxy-5-ene- and the 3-keto-4-ene structures of the ring A. Such structures have not been reported for other organisms or described as short-living intermediates [15]. Three gene clusters in the *S. hirsuta* genome have been found to encode the enzymes putatively related to the sterol and bile acid degradation pathways. As follows from a published bioinformatics search, the presence of steroid catabolism genes in the genome of thermophilic actinobacteria is not a common phenomenon.

Key genes related to the oxidative 9(10)-*seco* pathway of steroid degradation have been revealed in the genomes of only 7 of 52 thermophilic/thermotolerant species examined: *Thermomonospora curvata* DSM 43183, *Amycolatopsis granulosa* DSM 45669, *Amycolatopsis methanolica* strain 239T, *Amycolatopsis thermalba* strain 50.9b, *Amycolatopsis ruanii* strain 49.3e, *Thermocatellispora tengchongensis* DSM 45615, and *Microbispora siamensis* NBRC 104113 [15]. 

The ability to carry out some reactions leading to modification of the steroid molecule is inherent in mesophilic and some thermophilic microorganisms. For example, transformation of hydrocortisone into a 16α-hydroxy-derivative is carried out by mesophilic *Streptomyces roseochromogenes* [18]. The moderately thermophilic soil bacteria *Geobacillus stearothermophilus* and *Geobacillus kaustophilus* have been reported to hydroxylate progesterone derivatives and testosterone at position C6 [19,20]. The extremely thermophilic bacterium *Sulfolobus solfataricus* (=*Calderiella acidophila* MT4), has been shown to reduce the 3-keto group as well as the Δ^4^-double bond in various steroidal ketones [21]. However, such reactions could be possible with the participation of enzymes that are not related to sterol catabolism pathway. Indeed, key genes encoding enzymes involved in oxidative degradation of sterols have not been found in the genomes of *Geobacillus kaustophilus* and *Parageobacillus thermoglucosidasius* [15].

In this study, cholesterol bioconversion by *S.hirsuta* VKM Ac-666 was studied using mineral and rich media, and expression of key genes related to different stages of cholesterol catabolism was evaluated in *S.hirsuta* VKM Ac-666^T^ in response to cholesterol, and peculiarities of sterol degradation by this thermophilic actinobacterium were revealed.

## 2. Results

In our previous work, the temperature dependence of bacterial growth was evaluated and a temperature of 45 °C was chosen as optimal for *S. hirsuta* VKM Ac-666 cultivation and cholesterol bioconversion [15]. In this study, (i) cholesterol was evaluated as the only growth substrate for the strain and (ii) culture growth and cholesterol bioconversion were evaluated in a rich medium. Due to filamentous growth, OD was difficult to use as a growth indicator, and a gravimetric method and colony forming units (CFU) calculation were used instead.

### 2.1. Cholesterol as a Growth Substrate

**Figure 1:**
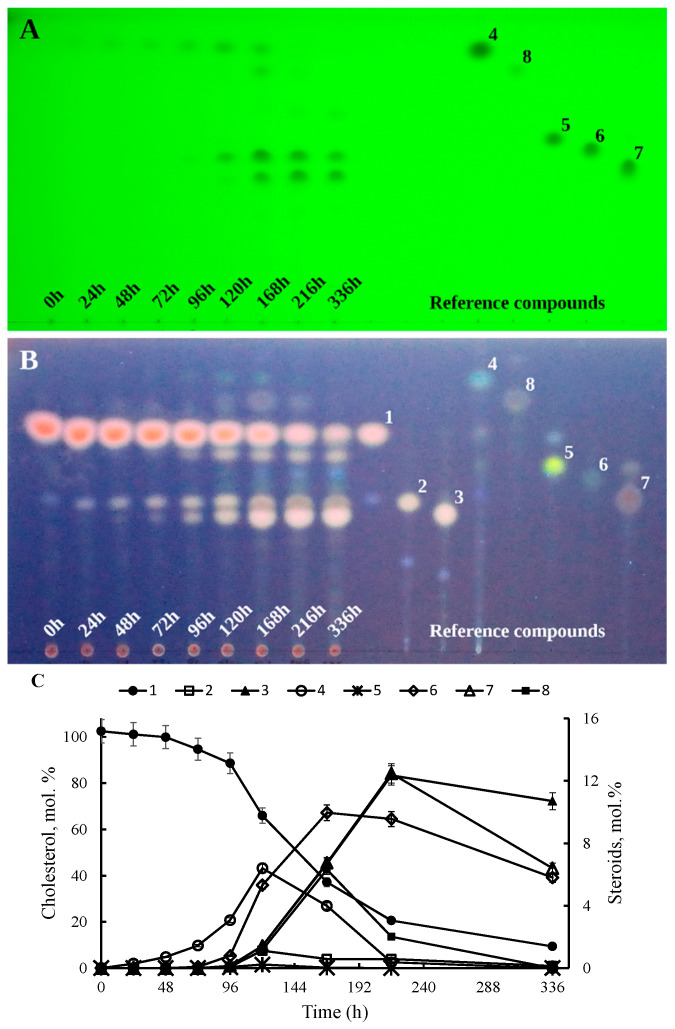
Cholesterol conversion by *S. hirsuta* in a mineral medium. (**A**) Identification under UV-light (254 nm). (**B**) TLC-plate staining with a MnCl_2_ solution followed by identification under UV light (365 nm). Each sample volume was 100 µL (**A**,**B**). (**C**) Kinetics of steroid products accumulation and cholesterol utilization (HPLC data). Designations: 1, cholesterol; 2, 26-hydroxycholesterol; 3, 3β-hydroxycholest-5-en-26-oic acid; 4, cholest-4-en-3-one; 5, 26-hydroxycholest-4-en-3-one; 6, 3-oxo-cholest-4-en-26-oic acid; 7, 3-oxo-cholesta-1,4-dien-26-oic acid; 8, cholesta-1,4-dien-3-one.

**Figure 2:**
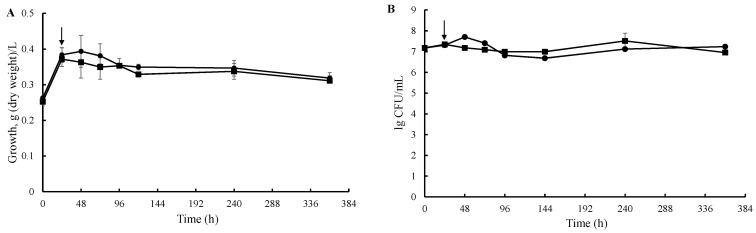
Culture growth in a mineral medium with cholesterol as the only carbon and energy source. (**A**) Biomass of *S. hirsuta* in a mineral medium in the presence of cholesterol (1.5 mM) (squares) or without steroid (circles, control) measured by dry weight; (**B**) CFU count in the dynamics of incubation of *S. hirsuta* in a mineral medium in the presence of cholesterol (1.5 mM) (squares) or without steroid (circles). Arrows indicate the addition of cholesterol.

When cholesterol (1) was used as the only growth substrate, 26-hydroxycholesterol (2), 3β-hydroxycholest-5-en-26-oic acid (3), cholest-4-en-3-one (4), 26-hydroxycholest-4-en-3-one (5), 3-oxo-cholest-4-en-26-oic acid (6), 3-oxo-cholesta-1,4-dien-26-oic acid (7), and cholesta-1,4-dien-3-one (8) were detected in the medium (Figure 1A–C). However, poor culture growth was observed on cholesterol as the sole source of carbon and energy, as evidenced by both biomass (dry cell weight, DCW) measurements and CFU counts (Figure 2A,B).

Interestingly, some growth retardation was observed after the addition of cholesterol to the medium. After 120 h of cultivation, the biomass remained the same in both variants for the next 240 h (Figure 2A). The CFU data were consistent with the results of biomass determination by dry weight (Figure 2B).

The *S. hirsuta* strain slowly utilized cholesterol in a mineral medium. The cholesterol conversion degree was more than 80%, and ~9 mol% cholesterol remained unconverted after 336 h of transformation (Figure 1C). Most likely, cholesterol was only partially used as a source of carbon, since another part (~20%) of it was converted into derivatives with a preserved side chain and steroid core (compounds 2–8 and others) after 336 h (Figure 1C).

Notably, the amounts of cholesterol derivatives reached their corresponding maximum levels after 120, 168, 216, and 336 h and were, respectively, ~6 mol% for cholest-4-en-3-one (4), ~10–mol% for 3-oxo-cholest-4-en-26-oic acid (6), ~12 mol% for 3-oxo-cholesta-1,4-dien-26-oic acid (7) (Figure 1C), and ~11 mol% for 3β-hydroxycholest-5-en-26-oic acid (3) (Figure 1C).

### 2.2. Cholesterol Bioconversion in a Rich Medium

**Figure 3:**
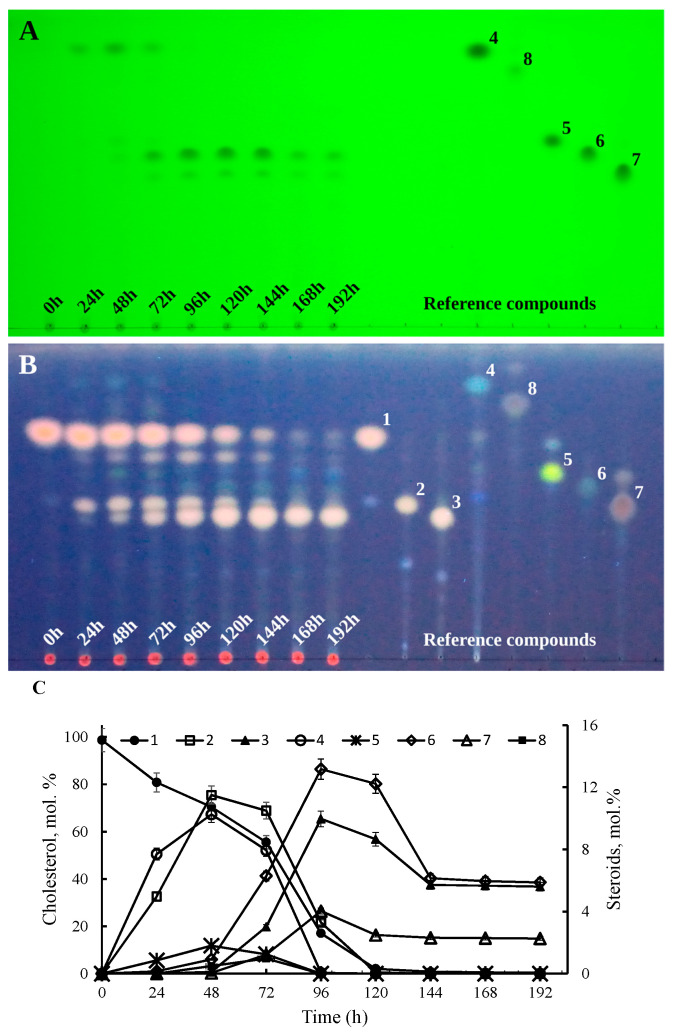
Cholesterol conversion by *S. hirsuta* in a rich (GSMY) medium. (**A**) Identification under UV light (254 nm). (**B**) TLC-plate staining with a MnCl_2_ solution followed by identification under UV light (365 nm). The sample volume to the spot was 50 µL (**A**,**B**). (**C**) Kinetics of steroid products accumulation and cholesterol utilization (HPLC data). Designations: 1, cholesterol; 2, 26-hydroxycholesterol; 3, 3β-hydroxycholest-5-en-26-oic acid; 4, cholest-4-en-3-one; 5, 26-hydroxycholest-4-en-3-one; 6, 3-oxo-cholest-4-en-26-oic acid; 7, 3-oxo-cholesta-1,4-dien-26-oic acid; 8, cholesta-1,4-dien-3-one.

When using a complex medium rich in nutrients (GSMY), cholesterol was almost fully converted by *S. hirsuta* after 144 h (Figure 3). After 24 h of cholesterol bioconversion, the main products were 26-hydroxycholesterol (2) and cholest-4-en-3-one (4), which were further metabolized to the corresponding 3β-5-ene-(3β-hydroxycholest-5-en-26-oic acid (3)) and 3-oxo-4-ene-(26-hydroxycholest-4-en-3-one (5), 3-oxo-cholest-4-en-26-oic acid (6), and 3-oxo-cholesta-1,4-dien-26-oic acid (7)) (Figure 3A–C). The residual cholesterol content did not exceed 1 mol% after 144 h of bioconversion (Figure 3C). Further, after 120 h, a significant decrease was observed in the contents of the main products, such as 3β-hydroxycholest-5-en-26-oic acid (compound 3, to ~6 mol%), 3-oxo-cholest-4-en-26-oic acid (compound 6, to ~6 mol%), and 3-oxo-cholesta-1,4-dien-26-oic acid (compound 7, to ~2 mol%) (Figure 3C).

The strain utilized cholesterol in the rich medium ~2 times faster than in the mineral medium. Comparisons of the bioconversion rates in mineral and rich media resulted in the conclusion that bioconversion patterns were similar, but the rates were different (Figure 1C vs. Figure 3C).

The steroid derivatives formed from cholesterol during its conversion by *S. hirsuta* via the action of the enzymes whose genes could be inducible by cholesterol.

**Figure 4:**
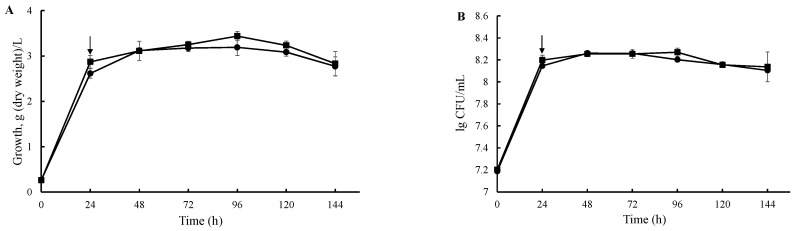
Growth of *S. hirsuta* in a rich (GSMY) medium. (**A**) *S. hirsuta* growth in a rich medium in the absence (circles) and presence of cholesterol (squares) (1.5 mM) measured by dry weight; (**B**) CFU count in the dynamics of incubation *S. hirsuta* in the rich medium in the presence of cholesterol (1.5 mM) (squares) or without steroids (circles). Arrows indicate the addition of cholesterol.

Analysis of the *S. hirsuta* growth in a rich medium in the absence (control) and presence of cholesterol revealed a pattern (Figure 4A) that was analogous to that in the mineral medium (Figure 2A). The CFU count data was similar to the results of biomass determination by dry weight (Figure 4A,B).

The results showed insignificant influence of cholesterol on the strain growth in the mineral or rich medium.

### 2.3. Quantitative Reverse Transcription–PCR (RT–qPCR)

**Figure 5:**
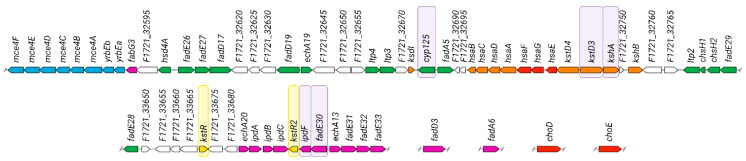
Organization of the *S. hirsuta* sterol catabolism gene cluster. Genes putatively involved in cholesterol catabolism are in green; genes related to the A/B-ring degradation are in red and orange; genes coding for C/D-ring degradation are in purple; genes coding for transport systems are in blue; and regulatory elements are in yellow. Five genes of interest are marked with light violet frames. The two transcriptional repressor genes *kstR* and *kstR2* are marked with yellow frames (adopted from [15]).

In this study, basal expression and transcriptional responses to cholesterol of five candidate genes from one large sterol catabolism gene cluster of the *S. hirsuta* genome (Figure 5) were investigated depending on the time of strain growth and cholesterol exposure. Expression of the candidate sterol catabolism genes was studied in the context of cholesterol decomposition and the growth phase of the strain, which might help to understand the mechanisms of sterol degradation and ecological adaption of the moderately thermophilic *S. hirsuta* strain.

The following genes were selected for the RT-qPCR expression analysis: *cyp125*, which is associated with the initial step in cholesterol side chain degradation [22,23]; *kstD3* and *kshA*, which encode enzymes responsible for key degradation steps of the steroid core [24,25]; and *fadE30* and *ipdF*, which are associated with the C/D ring degradation pathway [26,27,28]. Cyp125 is responsible for C26-hydroxylation of the terminal methyl group of cholesterol/cholestenone; KstDs and KshAs are the key enzymes for the ring B opening in the so-called 9(10)-*seco* pathway; IpdF is responsible for reduction of the C5-oxo-group to hydroxyl of the KstR2 inducer molecule HIP-CoA [1]; and FadE30 is involved in dehydrogenation of 5-OH-HIP-CoA [29] (Figure 5). The 16S rRNA gene was used as a reference gene.

**Figure 6:**
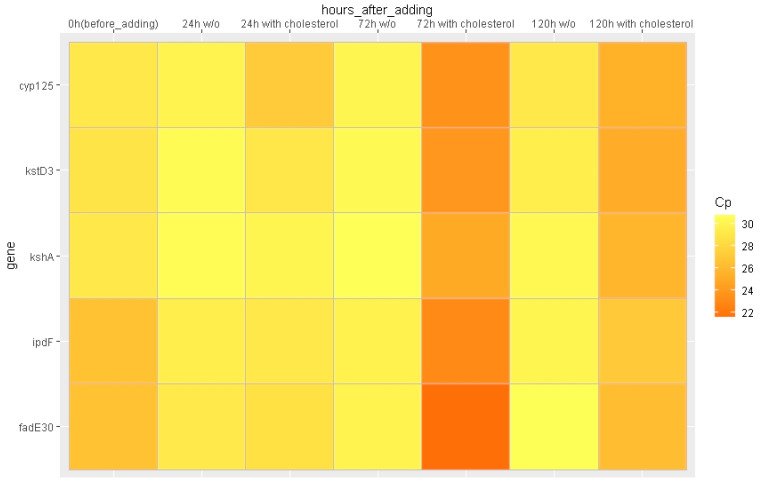
Heat map. The X-axis represents the sampling time for control and variant with cholesterol; the Y-axis represents the genes of interest. Crossing point (Cp) shows the cycle number for each gene of interest as normalized to the Cp of the 16S rRNA. Lower Cp values (higher expression) are brighter colors.

First of all, in order to determine whether the genes of interest (*cyp125*, *kstD3*, *kshA*, *ipdF*, and *fadE30*) function in the conditions under study, we analyzed the contents of their mRNAs in *S. hirsuta* cell samples taken at 0, 24, 72, and 120 h of incubation with cholesterol in the rich medium. The mRNAs of all five genes were detected in all samples, and their largest amounts were observed in the 72 h sample (Figure 6). Subsequently, changes in mRNA amount were evaluated only for 24- and 72-h samples in the rich medium.

**Figure 7:**
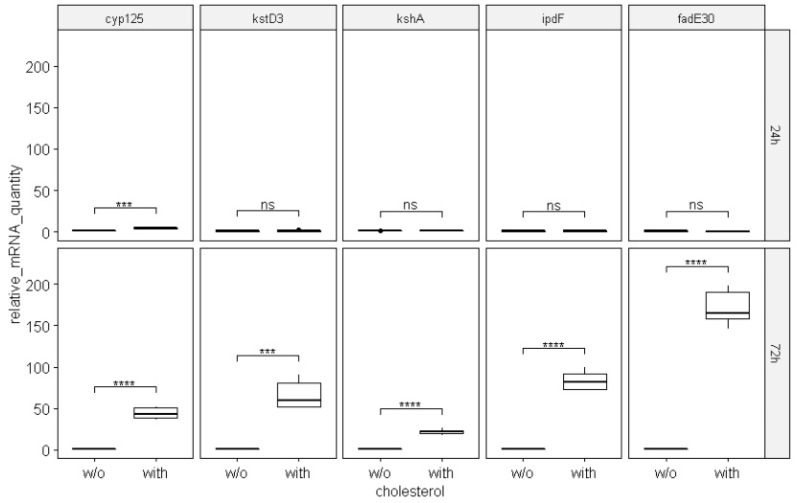
Relative mRNA quantities of the five genes of interest (normalized to the 16S rRNA amount) at 24 and 72 h of cholesterol induction. Designations: w/o, without; ns, *p* > 0.05; (***), *p* ≤ 0.001; (****), *p* ≤ 0.0001.

As follows from the data presented for the *cyp125* gene in Figure 6 and Figure 7, the relative mRNA amount increased 4.12 and 43.9 times in *S. hirsuta* cells cultivated in the presence of cholesterol for 24 h and 72 h, respectively.

For the genes *kstD3*, *kshA*, *ipdF*, and *fadE30*, no change in mRNA content was observed in *S. hirsuta* cells cultivated in the presence of cholesterol for 24 h. However, the amounts of their mRNAs increased statistically significantly after 72 h cholesterol exposure (Figure 7).

Upregulation levels of gene expression with cholesterol induction varied greatly among the genes of interest. Thus, there was only a 10.6-fold increase in expression of *cyp125* from 24 h to 72 h of cholesterol exposure. However, there were a 21.3-fold increase in *kshA* and 64.2-, 82-, and 169.3-fold increases in *kstD3*, *ipdF*, and *fadE30* expression, respectively, from 24 h to 72 h of cholesterol exposure. Based on these data, it can be assumed that the *cyp125* gene is more sensitive to cholesterol exposure during the initial 24 h than the other four genes of interest. Expression of *kshA*, *kstD3*, *ipdF*, and *fadE30* is greater after 72 h of cholesterol exposure.

Interestingly, the relative mRNA amount decreased for all genes under study throughout the subsequent cholesterol exposure from 72 to 120 h, but not to the same extent (Figure 6). The lowest relative mRNA amount corresponded to *ipdF*, and the greatest, to *kstD*.

### 2.4. High-Throughput mRNA Sequencing

An accumulation of two main cholesterol derivatives, 26-hydroxycholesterol and cholest-4-en-3-one, after 24 h of strain incubation with cholesterol in the rich medium (Figure 3C) indicated that the *S. hirsuta* enzymes that simultaneously and independently carry out 26-hydroxylation and transformation of the 3β-OH-5-ene- into the 3-oxo-4-ene-fragment of the cholesterol molecule, respectively, could be induced by cholesterol or one or more of its derivatives. It is known that cholesterol degradation in actinobacteria is regulated by transcriptional repressors of the TetR type, KstR and KstR2 [30,31]. Since 3-oxo-cholest-4-en-26-oic acid and its derivatives may regulate the transcription factor KstR [32] in actinobacteria, the finding of the structurally similar compounds 26-hydroxycholesterol and cholest-4-en-3-one among cholesterol degradation intermediates of the actinobacterial thermophile allowed us to suggest that comparative transcriptome profiling at 24 h of cholesterol exposure would shed light on the induction process in *S. hirsuta*. Given that oxidative degradation of cholesterol proceeds rapidly in some mesophilic actinobacteria and that, in contrast, a rather long lag period is observed in the process of cholesterol oxidation by the thermophilic *S. hirsuta* strain, we planned to record a clear picture of the sequential induction of genes related to the sterol degradation pathway at its early steps.

Based on these considerations, we decided to conduct whole-transcriptome analysis of *S. hirsuta* cells grown in the presence or absence of cholesterol for 24 h in the rich medium. The total number of reads, the percentage of reads mapping to the rRNA genes, and links to the SRA repository in NCBI for experimental variants are summarized in Table 1.

To identify the genes upregulated in response to cholesterol, differential gene expression was calculated in pairs of control and corresponding cholesterol-exposed samples. The total number of genes that increased in expression is shown in Table 2.

Among 28 *S. hirsuta* genes that significantly (from 3.03 to 40.83 times) increased in expression, only one gene, *cyp125* (*F1721_32680*), belonged to genes related to the sterol degradation pathway (Supplementary Table S1) [15].

Activities of other enzymes are most likely not associated with degradation of sterols. They could code for housekeeping proteins involved in regulating a variety of biological processes. Possible examples include transport and various signaling (*F1721_03575*); lipid export to the outer membrane through the inner membrane using ATP (*F1721_08445*); export of lipid components through the cell membrane (*F1721_04135*); pleiotropic regulation of carbon catabolite repression (*F1721_06445*); transport coordination of genes expression necessary for adaptation (*F1721_08455*); hydrolysis of a wide range of substrates bearing amide or ester functional groups (*F1721_08470*); control of gene expression by regulating the initiation or extension of transcription (*F1721_08460*); the ATP-binding cassette (ABC) transporters (*F1721_08450*); mediation of protein–protein interactions and assembly of multiprotein complexes (*F1721_15540*); hydrolysis of a wide range of substrates, such as beta-lactams, DNA or RNA (*F1721_15545*); 1′4-condensation between 5-carbon isoprene units (*F1721_22945*); methylation of proteins, small molecules, lipids, and nucleic acids using SAM as a cofactor (*F1721_27890*); metal ions resistance (*F1721_28715*); and a role of carrier proteins (*F1721_06460*).

The *F1721_15555* protein could be mentioned as potentially involved in metabolism of steroids (Table 2). A 2.9-fold increase was observed in expression of the *F1721_32655* gene in response to cholesterol. Other actinobacteria bearing steroid catabolism gene clusters have *F1721_32655* homologs: Rv3521 (in *M. tuberculosis* H37Rv) and *RHA1_RS22865* (in *R. jostii* RHA1) [15], which are close to the aldol lyase gene *ltp4* in the corresponding genomes. The products encoded by the *Rv3521* and *RHA1_RS22865* genes are known to be hypothetical conserved proteins. The product of the *S. hirsuta F1721_32655* gene potentially could bind to DNA, like KstR.

## 3. Discussion

**Figure 8:**
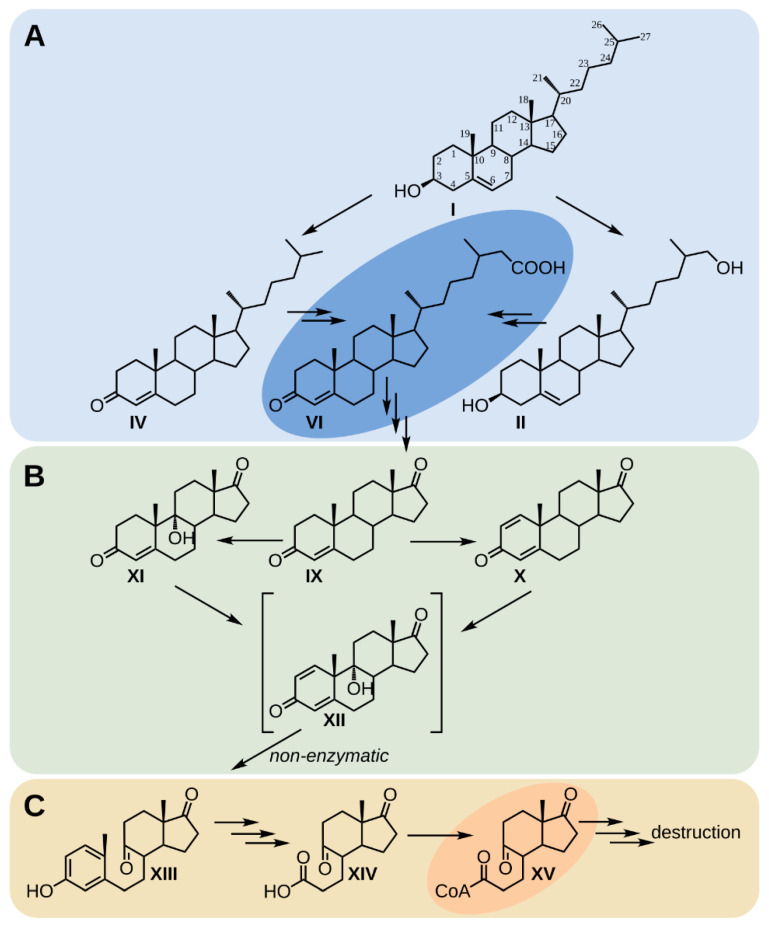
Principal scheme of cholesterol catabolism by mesophilic actinobacteria. (**A**) Dehydrogenation and isomerization of 3β-ol-5-ene to 3-keto-4-ene moiety in the steroid core A-ring and degradation of the sterol side chain to C19-steroid. (**B**) Steroid core B-ring opening via 9α-hydroxylation and 1(2)-dehydrogenation. (**C**) Steroid core degradation via the 9(10)-*seco* pathway. I, cholesterol; II, cholest-5-ene-3β,26-diol (26-Hydroxycholesterol); IV, cholest-4-en-3-one; VI, 3-oxo-cholest-4-en-26-oic acid, a KstR effector [32]; IX, androst-4-ene-3,17-dione (AD); X, androsta-1,4-diene-3,17-dione (ADD); XI, 9α-hydroxyandrost-4-ene-3,17-dione (9α-hydroxy-AD); XII, 9α-hydroxyandrosta-1,4-diene-3,17-dione (unsTable 9α-hydroxy-ADD); XIII, 3β-hydroxy-9,10-*seco*-androsta-1,3,5(10)-triene-9,17-dione (3-HSA); XIV, 9,17-dioxo-1,2,3,4,10,19-hexanorandrostan-5-oic acid (DOHNAA) or 3a*α*-H-4*α*-(3′-propanoate)-7a*β*-methylhexahydro-1,5-indadione (HIP); XV, 9,17-dioxo-1,2,3,4,10,19-hexanorandrostan-5-oyl-CoA (HIP-CoA), a KstR2 effector.

Degradation of sterols by actinobacteria is a complex process that includes cascades of enzymatic reactions [1,6] (Figure 8). In the last two decades, sterol catabolism has been intensively studied mainly in mesophilic actinobacteria, such as *Gordonia cholesterolivorans* Chol-3T [33], *Gordonia neofelifaecis* NRRL B-59395 [34], *M. tuberculosis* H37Rv [35,36], *Rhodococcus jostii* RHA1 [24], *Mycolicibacterium smegmatis* mc^2^ 155 [30], and *Nocardioides simplex* VKM Ac-2033D [37], while little is known about the representatives of thermophilic actinobacteria.

It has been shown that cholesterol oxidative degradation in mesophilic actinobacteria is controlled by two TetR-type transcriptional repressors, KstR and KstR2 [30,31,38,39]. KstR controls expression of genes whose products are involved in catabolism and responsible for activating the initial uptake of cholesterol, which is followed by β-oxidation of the cholesterol aliphatic side chain and opening and removal of the steroidal rings A and B [30,38,39]. KstR2 controls expression of cholesterol catabolic genes responsible for additional elements of the pathway that involve degradation of both the steroid C and D rings. 3-Oxo-cholest-4-en-26-oic acid and CoA-derivatives of 3-oxo-cholest-4-en-26-oic and 3-oxo-cholest-4-en-24-oic acids have been established to be KstR effectors [32,40]. In addition, hexahydro-indanedione propanoate-CoA (HIP-CoA) has been demonstrated to act as an efficient inducer of KstR2 [41].

Known microbial sterol degradation begins with C26-hydroxylation of the sterol alkyl side chain and/or modification of the A ring of the steroid core by converting 3β-hydroxy-5-ene- into a 3-oxo-4-ene-structure [1,42]. These two processes can occur simultaneously. Indeed, independent and simultaneous conversion of cholesterol into C26-hydroxylated 3β-hydroxy-5-ene- and 3-oxo-4-ene- corresponding derivatives has been clarified previously in the case of *S. hirsuta* [15].

As shown in this study, cholesterol is a poor growth substrate for *S. hirsuta.* The strain transforms cholesterol to form corresponding derivatives with the preserved side chain, such as cholest-4-en-3-one or 26-hydroxylated steroids. Much higher strain growth and cholesterol conversion/degradation rates were observed when a rich medium was used instead of a mineral one.

Nevertheless, the rate of cholesterol bioconversion by *S. hirsuta* is significantly inferior to the rates reported for mesophilic actinobacteria, such as *Mycobacterium* [34,43], *Mycolicibacterium* [39], *Rhodococcus* [44], *Gordonia* [34,39], and *Nocardioides* [37].

The fact that cholesterol bioconversion proceeds rapidly in mesophilic actinobacteria and slowly in a thermophilic actinobacterial strain prompted us to study the transcriptomic profiles of *S. hirsuta* cells grown with and without cholesterol in the GSMY medium at an early stage of sterol bioconversion.

The apparently longer duration of cholesterol bioconversion by *S. hirsuta* cells compared to mesophilic actinobacteria makes it possible to identify the early intermediates (or products) of cholesterol bioconversion. Surprisingly, whole transcriptome profiling revealed that, with the exception of c*yp125*, the vast majority of the genes previously described as cholesterol inducible did not increase their expression in response to cholesterol.

**Figure 9:**
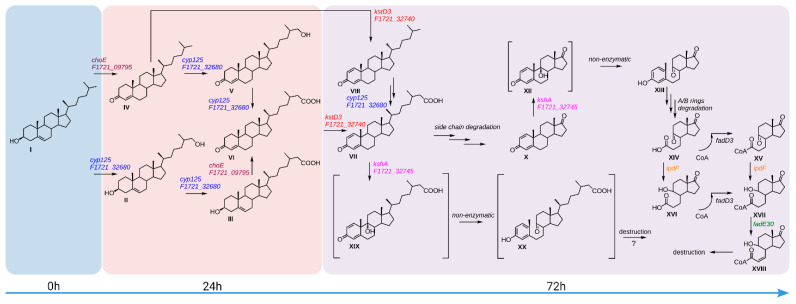
A scheme of the proposed involvement of the five genes of interest in cholesterol bioconversion by *S. hirsuta*. I, cholesterol; II, cholest-5-ene-3β,26-diol (26-hydroxycholesterol); III, 3β-hydroxy-cholest-5-en-26-oic acid; IV, cholest-4-en-3-one; V, 26-hydroxy-cholest-4-en-3-one; VI, 3-oxo-cholest-4-en-26-oic acid; VII, 3-oxo-cholesta-1,4-dien-26-oic acid; VIII, cholesta-1,4-dien-3-one; X, androsta-1,4-diene-3,17-dione (ADD); XII, 9α-hydroxyandrosta-1,4-diene-3,17-dione (unsTable 9α-hydroxy-ADD); XIII, 3β-hydroxy-9,10-seco-androsta-1,3,5(10)-triene-9,17-dione (3-HSA); XIV, 9,17-dioxo-1,2,3,4,10,19-hexanorandrostan-5-oic acid (DOHNAA) or 3aα-H-4α-(3′-propanoate)-7aβ-methylhexahydro-1,5-indadione (HIP); XV, 9,17-dioxo-1,2,3,4,10,19-hexanorandrostan-5-oyl-CoA (HIP-CoA); XVI, 9-hydroxy-17-oxo-1,2,3,4,10,19-hexanorandrostan-5-oic acid or 3aα-H-4α(3′-propanoate)-5α-hydroxy-7aβ-methylhexahydro-1-indanone (5-OH-HIP); XVII, 9-hydroxy-17-oxo-1,2,3,4,10,19-hexanorandrostan-5-oyl-CoA (HIP-CoA); XVIII—9-Hydroxy-17-oxo-1,2,3,4,10,19-hexanorandrost-6-ene-5-oyl-CoA (5-OH-HIPE-CoA); XIX, 9α-hydroxy-3-oxo-cholesta-1,4-dien-26-oic acid; XX, 3β-hydroxy-9-oxo-9,10-seco-cholesta-1,3,5(10)-triene-26-oic acid.

*Cyp125* encodes steroidal cytochrome P450 26(27)-monooxygenase, which catalyzes hydroxylation of the terminal methyl group of cholesterol/cholestenone to form the corresponding 26(27)-alcohols at early steps of sterol side chain degradation in actinobacteria [23]. Cyp125 (Cytochrome P450) is additionally responsible for the oxidation of 26-hydroxylated derivatives of cholesterol/cholestenone to corresponding C26-carboxylic acids [24,45]. In the strain under study, Cyp125 is most likely responsible for the production of 26-hydroxycholestenone; 26-hydroxycholesterol; and 3-oxo-cholest-4-ene-26-oic, 3-oxo-cholesta-1,4-diene-26-oic, and 3β-hydroxycholest-5-ene-26-oic acids from the corresponding precursors (Figure 9) because no orthologues of the *cyp124* and *cyp142* genes have been identified in the *S. hirsuta* genome [15].

However, the initial 24 h absence of any transcriptional response to cholesterol exposure from genes of the cholesterol catabolism cluster in *S. hirsuta* is consistent with some published data on mesophilic bacteria. For example, a differential expression analysis of *M. tuberculosis* H37Rv genes in the presence of cholesterol has demonstrated the greatest increase in expression for *cyp125* and *kshB* with virtually no response of the cholesterol import system *mce4* [43]. In contrast, expression of the steroid transporter *mce4* locus has been induced with cholesterol in *R. jostii* RHA1 [35,46], *M. smegmatis* mc^2^ 155 [39], and *N. simplex* [47].

Cholesterol microbial conversion to cholest-4-en-3-one is known to be carried out by cholesterol oxidases (ChoE) [48] and/or 3β-hydroxysteroid dehydrogenases (3β-HSD) [42]. Typically, either ChoE or 3β-HSD is active in every particular strain [39]. Mycobacterial species contain intracellular 3β-HSD [39], while some representatives of the *Rhodococcus* genus [48] and a *N. simplex* strain are characterized by the presence of secreted ChoEs [49,50].

The *choE* gene (*F1721_09795*) is outside the sterol catabolism clusters in the *S. hirsuta* genome [15] and was not upregulated in response to cholesterol, though expression of its *N. simplex* counterpart (*KR76_09550*) [49,50] has been reported to increase 10-fold and 13-fold in response to phytosterol and cholesterol, respectively [37,47]. Notably, the *choE* gene is outside the steroid catabolism clusters and possesses a putative KstR binding site in *N. simplex* [51]. It is also of interest that the important gene coding for 3β-HSD (*Rv1106c*) does not appear to be induced by cholesterol in *M. tuberculosis* H37Rv [43,52]. It is possible that the formation of 3-keto-4-ene steroids by the thermophilic *S. hirsuta* strain is due to high basal expression of another important enzyme, cholesterol oxidase encoded by *choE* (*F1721_09795*), because no gene coding for 3β-HSD has been revealed in its genome.

Despite the recorded formation of cholest-4-en-3-one and 26-hydroxycholesterol from cholesterol, no increase in the expression of *kstR*, *choE*, and or *mce4* genes was detected in response to cholesterol exposure of *S. hirsuta* cells for 24 h. Probably, the *mce4* genes could not be induced at the first stage of the metabolic response in *S. hirsuta*, but further incubation would increase the expression of the transporter genes.

It is possible to conclude that, in the absence of the induction of the genes *mce4* and *kstR* upon exposure to cholesterol for 24 h, the increase in expression of the *cyp125* gene is an important adaptive mechanism in the sterol-decomposing moderately thermophilic *S. hirsuta* strain.

Thus, as a result of cholesterol penetration into *S. hirsuta* cells, 26-hydroxylation promoted by Cyp125 occurred within 24 h to ensure the accumulation of 26-hydroxycholesterol and 3β-hydroxycholest-5-en-26-oic acid. Cholest-4-en-3-one detected in this work could also be hydroxylated at C26 with the formation of 26-hydroxycholest-4-en-3-one and 3-oxo-cholest-4-en-26-oic acid. Generally, Cyp125 (Cytochrome P450) and ChoE are important enzymes for synthesis of the KstR effector 3-oxo-cholest-4-en-26-oic acid (Figure 8) upon bioconversion of cholesterol.

Analysis of the gene arrangement in the *S. hirsuta* genome revealed that *cyp125* is not a part of the *fadA5* operon, based on the fact that *cyp125* and *fadA5* are on the opposite DNA strands (“head to head”). The *cyp125* gene could have its own promoter and, possibly, its own regulation. Thus, the genes of the sterol catabolism cluster are linked functionally, but not transcriptionally in the *S. hirsuta* genome.

In this work, activities of genes related to different stages of sterol catabolism in the thermophilic *S. hirsuta* strain was studied for the first time by RT–qPCR during the process of cholesterol bioconversion. Differential expression of five genes of interest: *cyp125* (*F1721_32680*), *kstD3* (*F1721_32740*), *kshA* (*F1721_32745*), *ipdF* (*F1721_33710*), and *fadE30* (*F1721_33715*) was observed in the stationary phase of cell growth in the GSMY medium following 72 h of cholesterol exposure.

The *kstD*, *kshA*, and *kshB* genes encode the corresponding KstD, KshA, and KshB enzymes involved in aerobic degradation of the steroid core in the so-called 9(10)-*seco* pathway, resulting in the formation of the chemically unsTable 1,4-diene-9α-hydroxy compound [1,6] (Figure 9). The location of *kstD3* (*F1721_32740*) and *kshA* (*F1721_32745*) side by side on the same DNA strand in the *S. hirsuta* genome (Figure 5) and the efficient mRNA synthesis in response to cholesterol make it possible to assume that the genes could be linked transcriptionally.

IpdF is an acyl-CoA dehydrogenase encoded by the *ipdF* gene. It is believed that the main function of this enzyme is reduction of the keto group to hydroxyl at C5 of HIP-CoA [1]. The FadE30 enzyme has been implicated in dehydrogenation of 5-OH-HIP-CoA and thus plays a significant role in steroid catabolism at the level of degradation of ethylhexahydroindanone propionate [29].

Expression of *ipdF* (*F1721_33710*) and *fadE30* (*F1721_33715*) in *S. hirsuta* cells was very high in response to cholesterol on evidence of RT–qPCR. Moreover, these genes are located one after the other on the same DNA strand (Figure 5).

Overall, the expression pattern of the sterol catabolism genes in the thermophilic *S. hirsuta* strain is similar to known examples of sterol catabolic gene expression in mesophilic actinobacteria. A significant increase in expression of the *kstD3* (*F1721_32740*) and *kshA* (*F1721_32745*) genes implicates the 9(10)-*seco* pathway in cholesterol degradation *in S. hirsuta* cells. Further destruction of the steroid core and the C and D rings is most likely due to the participation of the products of the *ipdF* (*F1721_33710*) and *fadE30* (*F1721_33715*) genes, which also showed a high level of expression in response to cholesterol. Past precedents for bioconversion of sterols have also confirmed that ring B opening can occur in the corresponding steroid intermediates, both containing and not containing an aliphatic side chain [53,54].

## 4. Materials and Methods

### 4.1. Materials

Cholesterol was obtained from Serva (Heidelberg, Germany); cholest-4-en-3-one, from Maybridge (Altrincham, UK); malt extract for microbiology and soluble starch were purchased from Sigma-Aldrich (St. Louis, MO, USA); randomly methylated β-cyclodextrin (MCD) was from Wacker-Chemie GmbH (München, Germany); yeast extract was from Difco (Franklin Lakes, NJ, USA). 26-Hydroxycholesterol, 3β-hydroxycholest-5-en-26-oic acid, 26-hydroxycholest-4-en-3-one, 3-oxo-cholest-4-en-26-oic acid, 3-oxo-cholesta-1,4-dien-26-oic acid, and cholesta-1,4-dien-3-one were obtained from the Laboratory of Bioengineering of Microbial Producers, Skryabin Institute of Biochemistry and Physiology of Microorganisms, Russian Academy of Sciences [15]. All other reagents were of the highest purity grade and were purchased from domestic commercial suppliers (Russia).

### 4.2. Microorganism, Cultivation, and Cholesterol Conversion

The strain of *Saccharopolyspora hirsuta* VKM Ac-666^T^ was obtained from the All-Russian Collection of Microorganisms (VKM).

The strain was cultured in flasks (750 mL) aerobically with constant stirring (200 rpm) at 45 °C. A seed culture was obtained using 50 mL of the GSMY medium [55], which contained (g/L): glucose, 7; soluble starch, 10; malt extract, 5; yeast extract, 4.5; and CaCO_3_, 0.05; pH 7.0–7.2. After 24 h of cultivation, the seed culture (5 mL) was added to 45 mL of the GSMY medium or a mineral medium, which contained (g/L): (NH_4_)_2_SO_4_, 3; KH_2_PO_4_, 1; K_2_HPO_4_, 5; MgSO_4_, 0.2; FeSO_4_, 0.01; and ZnSO_4_, 0.002; pH 7.0. After 24 h of inoculation, cholesterol was added as a solution with MCD (at a molar cholesterol: MCD ratio of 1:5) to a final concentration of 1.5 mM to both of the media. A corresponding MCD solution without cholesterol was added to the control. Cholesterol bioconversions were carried out aerobically (200 rpm) at 45 °C for 192 h or 336 h in the GSMY or mineral medium, respectively. The experiments were carried out in three replicates.

### 4.3. Isolation of Total RNA from S. hirsuta Cells for RNA-Seq

A *S. hirsuta* culture was grown in the GSMY medium as described in 4.2 *Microorganism*, *cultivation*, *and cholesterol conversion*. Aliquots (14 mL) of cultures grown with and without cholesterol were withdrawn at 24 h of cholesterol addition and centrifuged at 7000 g, 4 °C for 30 min. Total RNA was isolated from the precipitate using a RNeasy Mini Kit (QIAGEN, Hilden, North Rhine-Westphalia, Germany). The RNA concentration was measured with a Qubit 3.0 fluorometer (Thermo Fisher Scientific, Waltham, MA, USA) and a Qubit RNA BR reagent kit (Thermo Fisher Scientific, Waltham, MA, USA). RNA quality was assessed by capillary electrophoresis using a Bioanalyzer 2100 (Agilent Technologies, Santa Clara, CA, USA). Ribosomal RNA was removed using a Ribo-Zero Plus rRNA Depletion kit (Illumina, San Diego, CA, USA) according to the manufacturer’s protocol. A library was prepared with a NEBNext Ultra II Directional RNA Library Prep kit for Illumina (New England Biolabs, Ipswich, MA, USA) according to the manufacturer’s recommendations. The library was sequenced on an Illumina NextSeq500 system (Illumina, San Diego, CA, USA) to obtain 76-bp single reads.

### 4.4. Computational Analyses

Read quality was controlled using FastQC [56]. Adapter sequences and low quality regions in raw reads were removed using Trimmomatic program version 0.39 [57]. A mapping of prepared reads to the *S. hirsuta* VKM Ac-666^T^ genome obtained previously (accession number DDBJ/ENA/GenBank VWPH01000000) [14] was carried out using the Bowtie2 program version 2.4.5 [58]; mapped reads were counted using the featureCounts program version 1.6.4 [59]. The DESeq package version 1.34.1 was used to evaluate the differential gene expression [60]. A gene was considered to have changed the expression level if padj was >0.05.

### 4.5. Preparation of Total RNA for RT–qPCR

A *S. hirsuta* culture was grown in the GSMY medium as described in 4.2 *Microorganism*, *cultivation*, *and cholesterol conversion*. Aliquots (1 mL) of cultures grown with and without cholesterol were withdrawn at 0, 24, 72, and 120 h of cholesterol addition and centrifuged at 7000 g, 4 °C for 30 min. Total RNA was isolated from 40 mg of raw biomass pretreated with 5% phenol in ethyl alcohol. The biomass was resuspended in 400 μL of the TE buffer (pH 8.0) supplemented with 20 mg/mL lysozyme (AppliChem GmbH, Darmstadt, Germany) and 350 μg/mL proteinase K (Thermo Fisher Scientific, Waltham, MA, USA), kept at 37 °C for 10 min, and then combined with 50 mg of glass beads (~100 mesh). After four cycles of shaking at 3000 rpm for 2 min and incubation for 10 min at 37 °C, a lysis solution from the Aurum total RNA mini kit (Bio-Rad, Irvine, CA, USA) supplemented with polyvinylpyrrolidone 40,000 (AppliChem GmbH, Darmstadt, Germany) was added to a final concentration of 6%. The cells were kept at room temperature with continuous stirring for 30 min and then centrifuged at 13,400 rpm for 10 min. The resulting supernatant was mixed with 70% isopropanol as recommended by the manufacturer of the Aurum total RNA mini kit (Bio-Rad, Irvine, CA, USA). Further isolation of total RNA was carried out in accordance with the manufacturer’s protocol. The RNA concentration was determined spectrophotometrically. The quality of preparations was assessed by agarose gel electrophoresis.

### 4.6. RT–qPCR

Specific primers were constructed using the Primer-BLAST tool (Table 3).

A kit containing SYBR Green (cat. no. R-402, Syntol, Moscow, Russia) was used for qPCR. The following temperature program was used for amplification: (1) 95 °C for 3 min, (2) 95 °C for 10 s, (3) 60 °C for 20 s, (4) 72 °C for 5 s; 40 cycles included steps (2–4). For each pair of primers, the amplification efficiency was determined from the slope of the log-linear portion of the calibration curve (using a series of ten-fold dilutions of the template DNA). The reaction specificity was confirmed by agarose gel electrophoresis.

Total RNA preparations were treated with DNase I (New England Biolabs, Ipswich, MA, USA). A RevertAid RT Reverse Transcription kit (Thermo Fisher Scientific, Clontech Laboratories, Inc., Mountainview, CA, USA) and random hexamer primers were used to synthesize the first cDNA strand. The total RNA preparation (100 ng) was added to the reaction. Genomic DNA (gDNA) contamination was quantified (1/E^(Cp«-RT»-Cp«+RT»)) and did not exceed 3%. According to BestKeeper version 1 [61], the most stable was the 16S rRNA gene (SD 0.12, CV 1.7%).

### 4.7. RT–qPCR Data Analysis

Data to construct the heat map were obtained using the average of two biological replicates. Experiments to determine the relative mRNA amount (differential gene expression) were performed in six biological replicates. The relative mRNA amount was determined using the Pfaffl formula [61]. RStudio Desktop 2022.02.1 + 461 was used for statistical data analyses. The Shapiro–Wilk test was used to check the normality of the data. Data comparison was performed using Student’s unpaired t-test.

### 4.8. Growth Estimations

Cultures of *S. hirsuta* were grown in the GSMY or mineral medium as described in 4.2 *Microorganism*, *cultivation*, *and cholesterol conversion*. The *S. hirsuta* biomass grown in the presence or absence of cholesterol in the GSMY or mineral medium was assayed by CFU and dry weight. For a CFU count, serial dilutions were plated on Petri dishes with the agar GSMY medium and CFUs were subsequently counted after 48 h of growth. For dry weight measurements, samples (45 mL) of the cultivation broth were centrifuged at 5600× *g* for 40 min and the cakes were washed twice with 45 mL of 10% (*w*/*v*) aqueous MCD to remove cholesterol and then twice with 45 mL of distilled water. The washed cells were dried at 85 °C [62]. The growth experiments were carried out in three replicates.

### 4.9. Thin Layer Chromatography (TLC)

Samples (0.5 mL) of the cultivation broth were taken and extracted with 1 mL of ethyl acetate. The extracts were applied to TLC plates (ALUGRAM SIL G/UV254, Macherey-Nagel, Düren, Germany) and developed in heptane:benzene:acetone (20:20:15, *v*/*v*/*v*). Steroids with 3-oxo-4-ene moiety were visualized under UV light (254 nm) using a hemiscope CN-15MC UV Darkroom (Vilber Lourmat, Collégien, France); cholesterol and its derivatives were assayed after staining the TLC plates with a MnCl_2_ solution [63] and heating at 105 °C for 5–10 min and visualized under UV light (365 nm).

### 4.10. High-Performance Liquid Chromatography (HPLC)

HPLC analyses were performed using reversed-phase HPLC on Agilent Infinity 1200 system (Agilent Technologies, Waldbronn, Germany ) with Symmetry column (250 × 4.6 mm, 5 μm) with precolumn Symmetry C18 (5 μm, 3.9 × 20 mm) (Waters, Milford, MA, USA) at 50 °C and flow rate 1 mL/min. For assays of steroids, two mobile phases (acetonitrile:water:trifluoracetic acid (60:40:0.02, *v*/*v*/*v*, gradient elution mode) and acetonitrile:2-propanol:water (50:45:5, *v*/*v*/*v*, isocratic elution mode) with UV-detection at 210/200 nm (for compounds with 3β-ol-5-ene configuration according to the mobile phase) and 240 nm (for compounds with 3-oxo-4-ene configuration) were used.

Retention times (Rt): cholesterol, Rt 10.3 min (mobile phase acetonitrile:2-propanol:water 50:45:5 *v*/*v*/*v*, λ 200 nm); 26-hydroxycholesterol, Rt 23.09 min (mobile phase acetonitrile:water:trifluoracetic acid (60:40:0.02 *v*/*v*/*v*, λ 210 nm); 3β-hydroxycholest-5-en-26-oic acid, Rt 19.4 min (mobile phase acetonitrile:water:trifluoracetic acid (60:40:0.02 *v*/*v*/*v*, λ 210 nm); cholest-4-en-3-one, Rt 8.9 min (mobile phase acetonitrile:2-propanol:water 50:45:5 *v*/*v*/*v*, λ 240 nm); cholesta-1,4-dien-3-one, Rt 7.2 min (mobile phase acetonitrile:2-propanol:water 50:45:5 *v*/*v*/*v*, λ 240 nm); 26-hydroxycholest-4-en-3-one, Rt 23.04 min (mobile phase acetonitrile:water:trifluoracetic acid (60:40:0.02 *v*/*v*/*v*, λ 240 nm); 3-oxo-cholest-4-en-26-oic acid, Rt 20.1 min (mobile phase acetonitrile:water:trifluoracetic acid (60:40:0.02 *v*/*v*/*v*, λ 240 nm); 3-oxo-cholesta-1,4-dien-26-oic acid, Rt 17.6 min (mobile phase acetonitrile:water:trifluoracetic acid (60:40:0.02 *v*/*v*/*v*, λ 240 nm).

## 5. Conclusions

The thermophilic actinobacterium *S. hirsuta* utilizes cholesterol as the only carbon and energy source with a very low rate. Using a complex organic medium is favorable for cholesterol oxidation by the strain. The overall pattern of C26 steroid degradation observed during cholesterol conversion by the organism was similar to that reported for mesophilic actinobacteria. The genes involved in different stages of steroid degradation 9(10)-*seco*-pathway were upregulated sequentially in response to cholesterol in *S. hirsuta* and were not transcriptionally linked. To the best of our knowledge, this is the first report on the molecular mechanism of steroid degradation in thermophilic actinobacteria.

## Figures and Tables

**Table 1:** Sequencing statistics and links to reads.

Samples	Total Number of Raw Reads	Number of Reads Cleared	Number of Reads Mapped to Known Genes	Link to the Reads in SRA
Cholesterol, 1st replicate	34,373,229	33,902,023	30,245,313	SRR20572576
Cholesterol, 2nd replicate	34,260,954	33,937,790	30,213,192	SRR20572575
Cholesterol, 3rd replicate	38,157,119	37,718,807	33,240,441	SRR20572574
Control, 1st replicate	35,094,302	34,828,140	31,071,528	SRR20572579
Control, 2nd replicate	38,247,896	37,916,929	33,889,794	SRR20572578
Control, 3rd replicate	34,796,757	34,497,968	30,738,130	SRR20572577

**Table 2:** Genes of *S. hirsuta* whose expression increased in response to induction with cholesterol at 24 h.

№	Locus Tag	Gene Differential Expression in Response to Cholesterol	Link to the Reads in SRA Protein (NCBI Annotation)
1	*F1721*_*08450*	40.83	ABC transporter ATP-binding protein
2	*F1721*_*15540*	39.82	Tetratricopeptide repeat protein
3	*F1721*_*15545*	36.23	MBL fold metallo-hydrolase
4	*F1721*_*08445*	26.54	FtsX-like permease family protein
5	*F1721_27895*	22.82	DUF418 domain-containing protein
6	*F1721*_*27890*	17.11	Class I SAM-dependent methyltransferase
7	*F1721*_*22945*	10.67	Polyprenyl synthetase family protein
8	*F1721*_*08470*	9.71	Amidohydrolase
9	*F1721*_*06460*	9.22	DUF4111 domain-containing protein
10	*F1721*_*15550*	8.65	Hypothetical protein
11	*F1721*_*22940*	8.30	VOC family protein
12	*F1721*_*08455*	6.79	Two-component sensor histidine kinase
13	*F1721*_*15555*	5.66	SDR family oxidoreductase
14	*F1721*_*08440*	5.40	Nitric oxide synthase oxygenase
15	*F1721*_*17570*	4.40	Trypsin-like serine protease
16	***F1721*** **_*32680***	**4.35**	**Cytochrome P450**
17	*F1721*_*15565*	4.08	LysR family transcriptional regulator
18	*F1721*_*04135*	3.82	YhgE/Pip domain-containing protein
19	*F1721*_*08460*	3.71	Response regulator transcription factor
20	*F1721*_*15560*	3.64	Low temperature requirement protein A
21	*F1721*_*03575*	3.58	PDZ domain-containing protein
22	*F1721*_*15575*	3.39	L-rhamnose mutarotase
23	*F1721*_*06455*	3.35	Hypothetical protein
24	*F1721*_*14690*	3.34	PspC domain-containing protein
25	*F1721*_*04140*	3.33	MMPL family transporter
26	*F1721*_*22935*	3.24	Hypothetical protein
27	*F1721*_*28715*	3.17	Metal-sensitive transcriptional regulator
28	F1721_06445	3.03	LacI family transcriptional regulator

**Table 3:** Genes of *S. hirsuta* whose expression was increased in response to induction with cholesterol for 24 h.

Gene	Primer Sequence	Amplicon Size	E	R2
Genes of interest				
*cyp125*	cyp152(Ac-666)RT714f cgagttcgggttcttcgtga cyp152(Ac-666)RT830r ttgtacagctcccactggtc	117	1.84	0.993
*kstD3*	kstD3(Ac-666)RT1f atgaccgcagacaggttcg kstD3(Ac-666)RT170r tggttgttggggatccagac	170	2.03	0.990
*kshA*	kshA(Ac-666)RT547f tacatccacttcgcgttccc kshA(Ac-666)RT741r gcccttgtactcgttccaca	195	1.85	0.994
*ipdF*	ipdF(Ac-666)RT590f tggtgatgcacgagaacctg ipdF(Ac-666)RT740r ccggtgaggtaggtcgagta	151	1.91	0.997
*fadE30*	fadE30(Ac-666)RT1116f gatccagcgcaacatcatcg fadE30(Ac-666)RT1175r cacggtcggctctccttg	60	1.83	0.999
Reference gene				
16s rRNA	16S(Ac-666)RT33f gggtaatctgccctgcact 16S(Ac-666)RT275r gattccccactgctgcctc	243	1.92	0.998

## Data Availability

The datasets generated for this study can be found in the NCBI Sequence Read Archive (https://www.ncbi.nlm.nih.gov/sra/SRR20572576, https://www.ncbi.nlm.nih.gov/sra/SRR20572575, https://www.ncbi.nlm.nih.gov/sra/SRR20572574, https://www.ncbi.nlm.nih.gov/sra/SRR20572579, https://www.ncbi.nlm.nih.gov/sra/SRR20572578, https://www.ncbi.nlm.nih.gov/sra/SRR20572577 (all URL accessed on 24 July 2022)).

## Supplementary Materials

The following supporting information can be downloaded at: https://www.mdpi.com/article/10.3390/ijms232416174/s1, Supplementary Table S1. Changes in the level of sterol catabolism genes in the *S. hirsuta* genome during transcriptomic profiling in response to cholesterol.
