# Supplementary material for: Insight into Different Stages of Steroid Degradation in Thermophilic Saccharopolyspora hirsuta VKM Ac-666T Strain
Source: Int J Mol Sci. 2022 Dec 18;23(24):16174. doi: 10.3390/ijms232416174 (PMC9782250; doi:10.3390/ijms232416174)
Supplement: Supplementary file 1 [file ijms-23-16174-s001.zip › ijms-2035512-supplementary.pdf]

International Journal of *Molecular Sciences*

MDPI

Supplementary Materials

Insight into Different Stages of Steroid Degradation in Thermophilic *Saccharopolyspora hirsuta* VKM Ac-666^{T} Strain

Tatyana Lobastova [1], Victoria Fokina [1,*], Irina Pozdnyakova-Filatova [2], Sergey Tarlachkov [1] and Marina Donova [1]

[1] Laboratory of Bioengineering of Microbial Producers, G.K. Skryabin Institute of Biochemistry and Physiology of Microorganisms, RAS, Federal Research Center "Pushchino Scientific Center for Biological Research of the Russian Academy of Sciences", Pushchino 142290, Russia

[2] Laboratory of Molecular Microbiology, G.K. Skryabin Institute of Biochemistry and Physiology of Microorganisms, RAS, Federal Research Center "Pushchino Scientific Center for Biological Research of the Russian Academy of Sciences", Pushchino 142290, Russia

* Correspondence: 2vvfokina@gmail.com

International Journal of Molecular Sciences

MDPI

Supplementary Table S1. Changes in the level of sterol catabolism genes in the *S. hirsuta* genome during transcriptomic profiling in response to cholesterol.

No.	Scaffold acc.	Locus tag	Protein acc.	Name/Function	fold Change	padj
				Mce4 transport system		
1.	VWPH01000020.1	*F1721_32550*	KAA5825886.1	*mce4F*	0.82	1.00E+00
2.	VWPH01000020.1	*F1721_32555*	KAA5825887.1	*mce4E*	1.22	1.00E+00
3.	VWPH01000020.1	*F1721_32560*	KAA5825888.1	*mce4D*	0.97	1.00E+00
4.	VWPH01000020.1	*F1721_32565*	KAA5825937.1	*mce4C*	0.87	1.00E+00
5.	VWPH01000020.1	*F1721_32570*	KAA5825889.1	*mce4B*	1.00	1.00E+00
6.	VWPH01000020.1	*F1721_32575*	KAA5825890.1	*mce4A*	1.00	1.00E+00
7.	VWPH01000020.1	*F1721_32580*	KAA5825891.1	*yrbEb*	0.91	1.00E+00
8.	VWPH01000020.1	*F1721_32585*	KAA5825892.1	*yrbEa*	1.38	1.00E+00
				Sterol side chain degradation		
9.	VWPH01000020.1	*F1721_32590*	KAA5825893.1	*fabG3*	1.56	1.00E+00
10.	VWPH01000020.1	*F1721_32595*	KAA5825894.1	aldehyde dehydrogenase	1.30	1.00E+00
11.	VWPH01000020.1	*F1721_32600*	KAA5825895.1	*hsd4A*	1.7	1.00E+00

12.	VWPH01000020.1	*F1721_32605*	KAA5825896.1	*fadE26*	0.92	1.00E+00
13.	VWPH01000020.1	*F1721_32610*	KAA5825897.1	*fadE27*	0.59	1.00E+00
14.	VWPH01000020.1	*F1721_32615*	KAA5825898.1	*fadD17*	1.08	1.00E+00
15.	VWPH01000020.1	*F1721_32620*	KAA5825899.1	alpha/beta hydrolase	0.89	1.00E+00
16.	VWPH01000020.1	*F1721_32625*	KAA5825900.1	MerR family transcriptional regulator	0.89	1.00E+00
17.	VWPH01000020.1	*F1721_32630*	KAA5825901.1	nitronate monooxygenase	1.27	1.00E+00
18.	VWPH01000020.1	*F1721_32635*	KAA5825902.1	*fadD19*	1.75	1.00E+00
19.	VWPH01000020.1	*F1721_32640*	KAA5825903.1	*echA19*	2.16	1.00E+00
20.	VWPH01000020.1	*F1721_32645*	KAA5825904.1	acyl-CoA synthetase	1.15	1.00E+00
21.	VWPH01000020.1	*F1721_32650*	KAA5825905.1	LLM class F420-dependent oxidoreductase	0.80	1.00E+00
22.	VWPH01000020.1	*F1721_32655*	KAA5825906.1	DNA-binding protein	1.93	7.88E-01
23.	VWPH01000020.1	*F1721_32660*	KAA5825907.1	*ltp4*	1.41	1.00E+00
24.	VWPH01000020.1	*F1721_32665*	KAA5825908.1	*ltp3*	1.38	1.00E+00
25.	VWPH01000020.1	*F1721_32670*	KAA5825909.1	F420-dependent LLM class oxidoreductase	1.18	1.00E+00
26.	VWPH01000020.1	*F1721_32675*	KAA5825910.1	*ksdI*	1.82	1.00E+00

27.	VWPH01000020.1	*F1721_32680*	KAA5825911.1	*cyp125*	4.35	1.22E-06
28.	VWPH01000020.1	*F1721_32685*	KAA5825912.1	*fadA5*	1.02	1.00E+00
29.	VWPH01000020.1	*F1721_32690*	KAA5825913.1	DUF4180 domain-containing protein	0.83	1.00E+00
			Ring A/B oxidation			
30.	VWPH01000020.1	*F1721_32695*	KAA5825938.1	helix-turn-helix domain-containing protein	1.02	1.00E+00
31.	VWPH01000020.1	*F1721_32700*	KAA5825914.1	*hsaB*	0.98	1.00E+00
32.	VWPH01000020.1	*F1721_32705*	KAA5825915.1	*hsaC*	1.22	1.00E+00
33.	VWPH01000020.1	*F1721_32710*	KAA5825916.1	*hsaD*	1.15	1.00E+00
34.	VWPH01000020.1	*F1721_32715*	KAA5825917.1	*hsaA*	1.23	1.00E+00
35.	VWPH01000020.1	*F1721_32720*	KAA5825918.1	*hsaF*	1.07	1.00E+00
36.	VWPH01000020.1	*F1721_32725*	KAA5825919.1	*hsaG*	1.30	1.00E+00
37.	VWPH01000020.1	*F1721_32730*	KAA5825920.1	*hsaE*	0.85	1.00E+00
38.	VWPH01000020.1	*F1721_32735*	KAA5825921.1	*kstD4*	1.27	1.00E+00
39.	VWPH01000020.1	*F1721_32740*	KAA5825922.1	*kstD3*	1.35	1.00E+00
40.	VWPH01000020.1	*F1721_32745*	KAA5825923.1	*kshA*	1.62	1.00E+00

41.	VWPH01000020.1	*F1721_32750*	KAA5825924.1	hypothetical protein	1.51	1.00E+00
42.	VWPH01000020.1	*F1721_32755*	KAA5825925.1	*kshB*	1.29	1.00E+00
				"*igr*-locus"		
43.	VWPH01000020.1	*F1721_32760*	KAA5825939.1	FAD-binding oxidoreductase	1.10	1.00E+00
44.	VWPH01000020.1	*F1721_32765*	KAA5825926.1	WYL domain-containing protein	0.90	1.00E+00
45.	VWPH01000020.1	*F1721_32770*	KAA5825927.1	*ltp2*	0.65	1.00E+00
46.	VWPH01000020.1	*F1721_32775*	KAA5825928.1	*chsH1*	0.88	1.00E+00
47.	VWPH01000020.1	*F1721_32780*	KAA5825929.1	*chsH2*	1.19	1.00E+00
48.	VWPH01000020.1	*F1721_32785*	KAA5825930.1	*fadE29*	1.40	1.00E+00
49.	VWPH01000024.1	*F1721_33645*	KAA5825036.1	*fadE28*	1.53	1.00E+00
50.	VWPH01000024.1	*F1721_33650*	KAA5825037.1	class I SAM-dependent methyltransferase	0.90	1.00E+00
51.	VWPH01000024.1	*F1721_33655*	KAA5825022.1	prenyltransferase	1.06	1.00E+00
52.	VWPH01000024.1	*F1721_33660*	KAA5825023.1	methyltransferase domain-containing protein	1.04	1.00E+00
53.	VWPH01000024.1	*F1721_33665*	KAA5825024.1	glycosyltransferase family 4 protein	1.11	1.00E+00
				Transcriptional regulator		

54.	VWPH01000024.1	*F1721_33670*	KAA5825025.1	*kstR*	1.57	5.72E-01
			Ring C/D degradation			
55.	VWPH01000024.1	*F1721_33675*	KAA5825026.1	MBL fold metallo-hydrolase	0.99	1.00E+00
56.	VWPH01000024.1	*F1721_33680*	KAA5825027.1	SDR family oxidoreductase	1.08	1.00E+00
57.	VWPH01000024.1	*F1721_33685*	KAA5825028.1	*echA20*	0.96	1.00E+00
58.	VWPH01000024.1	*F1721_33690*	KAA5825029.1	*ipdA*	1.20	1.00E+00
59.	VWPH01000024.1	*F1721_33695*	KAA5825030.1	*ipdB*	1.42	1.00E+00
60.	VWPH01000024.1	*F1721_33700*	KAA5825038.1	*ipdC*	0.96	1.00E+00
61.	VWPH01000024.1	*F1721_33705*	KAA5825039.1	*kstR2*	1.09	1.00E+00
62.	VWPH01000024.1	*F1721_33710*	KAA5825031.1	*ipdF*	0.80	1.00E+00
63.	VWPH01000024.1	*F1721_33715*	KAA5825032.1	*fadE30*	0.97	1.00E+00
64.	VWPH01000024.1	*F1721_33720*	KAA5825033.1	*echA13*, enoyl-CoA hydratase	1.24	1.00E+00
65.	VWPH01000024.1	*F1721_33725*	KAA5825040.1	*fadE31*	0.99	1.00E+00
66.	VWPH01000024.1	*F1721_33730*	KAA5825034.1	*fadE32*	0.74	1.00E+00
67.	VWPH01000024.1	*F1721_33735*	KAA5825035.1	*fadE33*	0.91	1.00E+00

Cholate degradation pathway						
68.	VWPH01000001.1	F1721_00675	KAA5838019.1	*hsaE3*	0.82	1.00E+00
69.	VWPH01000001.1	*F1721_00680*	KAA5838020.1	*hsaG3*	0.95	1.00E+00
70.	VWPH01000001.1	*F1721_00685*	KAA5838021.1	*hsaF3*	0.85	1.00E+00
71.	VWPH01000001.1	*F1721_00690*	KAA5838022.1	*casA*	0.85	1.00E+00
72.	VWPH01000001.1	*F1721_00695*	KAA5838023.1	*hsaD3*	1.57	9.85E-01
73.	VWPH01000001.1	*F1721_00700*	KAA5838439.1	*kstR3*	0.90	1.00E+00
74.	VWPH01000001.1	*F1721_00705*	KAA5838024.1	SDR family NAD(P)-dependent oxidoreductase	1.4	1.00E+00
75.	VWPH01000001.1	*F1721_00710*	KAA5838025.1	*kstD2*	0.71	1.00E+00
76.	VWPH01000001.1	*F1721_00715*	KAA5838026.1	*kstD1*	0.74	1.00E+00
77.	VWPH01000001.1	*F1721_00720*	KAA5838027.1	SDR family oxidoreductase	0.83	1.00E+00
78.	VWPH01000001.1	*F1721_00725*	KAA5838028.1	*kshA*	0.60	1.00E+00
79.	VWPH01000001.1	*F1721_00730*	KAA5838029.1	hypothetical protein	0.66	1.00E+00
80.	VWPH01000001.1	*F1721_00735*	KAA5838030.1	*kshB*	0.58	1.00E+00
81.	VWPH01000001.1	*F1721_00740*	KAA5838031.1	*ksdI*	1.17	1.00E+00

82.	VWPH01000001.1	*F1721_00745*	KAA5838032.1	*hsaB3*	0.74	1.00E+00
83.	VWPH01000001.1	*F1721_00750*	KAA5838440.1	SDR family oxidoreductase	0.94	1.00E+00
84.	VWPH01000001.1	*F1721_00755*	KAA5838033.1	*hsaA3*	0.91	1.00E+00
85.	VWPH01000001.1	*F1721_00760*	KAA5838034.1	*hsaC3*	0.82	1.00E+00
86.	VWPH01000015.1	*F1721_28740*	KAA5828429.1	class I SAM-dependent methyltransferase	1.03	1.00E+00
87.	VWPH01000015.1	*F1721_28745*	KAA5828430.1	*casA*	0.91	1.00E+00
88.	VWPH01000015.1	*F1721_28750*	KAA5828431.1	*casC/chsE3*	1.28	1.00E+00
89.	VWPH01000015.1	*F1721_28755*	KAA5828432.1	*casE*	1.10	1.00E+00
90.	VWPH01000015.1	*F1721_28760*	KAA5828433.1	*3α-hsd*	1.15	1.00E+00
91.	VWPH01000015.1	*F1721_28765*	KAA5828434.1	*casH*	1.32	1.00E+00
92.	VWPH01000015.1	*F1721_28770*	KAA5828435.1	*casI*	2.24	1.00E+00
93.	VWPH01000001.1	*F1721_02365*	KAA5838304.1	*casE*	0.75	1.00E+00
94.	VWPH01000001.1	*F1721_02405*	KAA5838310.1	*casG*	0.76	1.00E+00
			HIP-CoA synthetase			
95.	VWPH01000019.1	*F1721_32060*	KAA5826116.1	*fadD3*	0.76	1.00E+00

			Thiolase			
96.	VWPH01000031.1	*F1721_34155*	KAA5824600.1	*fadA6*	0.94	1.00E+00
			Cholesterol oxidases			
97.	VWPH01000006.1	*F1721_14655*	KAA5833518.1	*choD*	0.73	1.00E+00
98.	VWPH01000004.1	*F1721_09795*	KAA5835081.1	*choE*	0.89	1.00E+00
			Transcriptional regulator			
99.	VWPH01000015.1	*F1721_28735*	KAA5828487.1	*kstR3*	0.98	1.00E+00
